# Computational investigation on the antioxidant activities and on the M^pro^ SARS-CoV-2 non-covalent inhibition of isorhamnetin

**DOI:** 10.3389/fchem.2023.1122880

**Published:** 2023-01-24

**Authors:** Maciej Spiegel, Giada Ciardullo, Tiziana Marino, Nino Russo

**Affiliations:** ^1^ Dipartimento di Chimica e Tecnologie Chimiche, Università della Calabria, Rende, Cosenza, Italy; ^2^ Department of Pharmacognosy and Herbal Medicines, Wroclaw Medical University, Wroclaw, Poland

**Keywords:** antioxidants, chemical equilibria, reaction mechanisms, kinetic constant, copper complexes, Fenton’s reaction, main protease inhibition

## Abstract

In the present work, we report a computational study on some important chemical properties of the flavonoid isorhamnetin, used in traditional medicine in many countries. In the course of the study we determined the acid-base equilibria in aqueous solution, the possible reaction pathways with the •OOH radical and the corresponding kinetic constants, the complexing capacity of copper ions, and the reduction of these complexes by reducing agents such as superoxide and ascorbic anion by using density functional level of theory Density Functional Theory. Finally, the non-covalent inhibition ability of the SARS-CoV-2 main protease enzyme by isorhamnetin was examined by molecular dynamics (MD) and docking investigation.

## 1 Introduction

A commonly accepted definition of oxidative stress is an imbalance between the production of reactive free radicals and the ability of the organism to inactivate thembefore their excessive production becomes harmful (Sies, 1985; Sies et al., 2017). In other words, it is defined when the action of oxidizing agents is not effectively counteracted by molecules present in our body that have antioxidant capacities.

Oxidative stress damages cells and organs, and is a direct or indirect cause of many conditions, ranging from cancer to atherosclerosis, neurodegenerative disorders (e.g., Alzheimer’s and others) and pulmonary diseases (Forman and Zhang, 2021). For these reasons, an enormous amount of scientific work in recent decades has concerned both the specific damage caused by oxidative stress and the chemical mechanisms of action underlying oxidative processes, as well as compounds capable of preventing them.

Considerable attention has been paid to understanding the structures, chemical-physical properties and mechanisms of action of both natural and synthetic antioxidant compounds (Leopoldini et al., 2011; Galano et al., 2016a; Apak et al., 2016). Many antioxidants originate from the plant kingdom (e.g., fruits, cereals, vegetables, and plants) and have been used in traditional medicinal systems of European, African, Asian, and American medicine for many centuries. In particular, flavonoid compounds of natural origin have proven to be powerful antioxidant systems, suitable for combating oxidative stress. Their actions are multidirectional, but the most important can be outlined as:

1) direct scavenging of free radicals, also known as primary antioxidation, which essentially consists of a reaction between a powerful free radical and an antioxidant with the formation of a new less active radical, and reaction can proceed until the formation of a neutral specie (Milenković et al., 2020). This reaction can occur through different mechanisms such as transferring a hydrogen atom or an electron or both from the antioxidant to the free radical (HAT and SET), or sequential proton-loss electron transfer (SPLET), or by bonding the radical to the structure of the antioxidant (RAF) (Galano et al., 2016b; Spiegel, 2022):

$$HAT:\;H_{n}A+R^{•}\;\to \;H_{n-1}A^{•}+RH$$

$$SET:\;H_{n}A+R^{•}\to \;H_{n}A^{+•}+R^{-}$$

$$RAF:\;H_{n}A+R^{•}\;\to \;{\left[H_{n-1}A-RH\right]}^{•}$$

$$SPLET:\;H_{n}A\;\to \;H_{n-1}A^{-}+H^{+}\;;\;H_{n-1}A^{-}+R^{•}\;\to \;H_{n-1}A^{•}+R^{-}$$

2) the capacity of the compound to chelate copper and iron ions responsible for the production of free radicals through the Fenton’s reaction. It is also referred to as a secondary antioxidant effect and is also relevant to the possible treatment of Alzheimer’s disease based on complexation of these metals (Sharma et al., 2013; Spiegel et al., 2022a):

$$Cu{\left(H_{2}O\right)}_{4}^{2+}+L^{m}\;\to \;{\left[Cu{\left(H_{2}O\right)}_{4}\;L^{m}\right]}^{2+}\;\left(m=0,-1,-2\right)$$

In addition, some antioxidants are effective inhibitor of a number of enzymes implicated in various diseases, including SARS-CoV-2 (Yu et al., 2012; Puttaswamy et al., 2020; Xu et al., 2020).

In this article, we report a theoretical (DFT) and computations (MD and docking) investigation that can contribute to a better understanding of the mechanisms of action of isorhamnetin antioxidant flavonoid (H_4_Iso), which contains hydroxyl groups in its backbone, which are the main structural elements on which the antioxidant capacity of polyphenols rely. Isorhamnetin is present in the juice of Chinese sea buckthorme (Pengfei et al., 2009), and can also be extracted from the fruits, flowers and leaves of *Ginkgo biloba*, *A. roxburghii*, and *Hippophae rhmnoides L.* (Teng and Luan, 2016; Gong et al., 2020). Furthermore, the great importance of isorhamnetin is due to the fact that it is a metabolite of quercetin, converted by the enzyme catechol-O-metyltransferase. This means that isorhamnetin concentrations in human plasma high, even when only quercetin is taken (Manach et al., 1997).

In order to highlight the performance of theoretical methods in reproducing and predicting antioxidant properties and related mechanisms in environments simulating physiological ones (aqueous and lipid), the paper will examine: 1) the acid-base equilibria in water solvent, 2) the reactions between the ^•^OOH radical and isorhamnetin, 3) the chelating capacity, and 4) the inhibition process of the SARS-CoV-2 main protease enzyme.

## 2 Methods and computational details

All the computations were performed using Density Functional Theory (DFT) implemented in Gaussian 09 code (Frisch et al., 2014) and following the QM-ORSA (Quantum Mechanics-Based Test for Overall Free Radical Scavenging Activity) computational protocol (Galano and Alvarez-Idaboy, 2013). We used the M06-2X (Zhao et al., 2006) exchange-correlation functional, the 6-311+G (d,p) basis set and the solvation model based on density (SMD) (Marenich et al., 2009), previously tested and applied to a series of molecules with antioxidant properties (Belcastro et al., 2006; Alberto et al., 2013; Ngo et al., 2019; Castaneda-Arriaga et al., 2020; Parise et al., 2021a; Reina et al., 2021; Spiegel et al., 2021; Spiegel, 2022). Pentylethanoate (ε = 4.7) and water (ε = 78.4) was selected to simulate the physiological relevant environments. The geometries of minima (reactants and products) and transition states (TS) located along the considered reaction pathways were optimized and characterized by computing vibrational frequencies and establishing the intrinsic reaction coordinates (IRC).

For the open-shell systems, the unrestricted procedure was used. The p*K*_a_ values and molar fractions for neutral and charged species were determined according to the methodology proposed and tested earlier (Galano et al., 2016a).

To compute kinetic rate constants conventional transition state theory ((Evans and Polanyi, 1935; Truhlar et al., 1996) was used, and for reactions close to the diffusion limit, the Collins–Kimball theory was applied (Collins and Kimball, 1949). For SET reactions, energy barriers were calculated adopting the Marcus theory (Marcus, 1993). The intrinsic reactivity indices such as bond dissociation energy (BDE), ionization potential (IP), proton affinity (PA) and proton desorption energy (PDE) were estimated under the adiabatic approximation with the following values of solvation enthalpies of H^+^ (ΔH(H^+^) = 1,055.7 kJ/mol) and electron (ΔH (e^−^) = 77.5 kJ/mol. (Markovic et al., 2016). To visualize the structures the MarvinSketch version 21.15.0 software (ChemAxon) was used.

The initial unbound structure of the SARS-CoV-2 main protease (M^pro^) has been obtained using the crystal structure of main protease bound to non-covalent inhibitor (PDB code 6W63) (https://www.wwpdb.org/pdb?id=pdb_00006w63, 2020), removing the inhibitor from the crystallographic structure and adding the hydrogen atoms by using H++ (Anandakrishnan et al., 2012) and to calculate the protonation states of titratable residues at pH 7.4. Both the protonation states of the His41 catalytic residue neutral, with the hydrogen on the N_δ_ and N_ε_ side chain have been considered. 300 ns MDs were performed, by using AMBER16 code (Case et al., 2017) and the FF14SB force field (Maier et al., 2021), for the two protonation states of the unbound M^pro^ considering a solvated orthorhombic box with a buffer of 10 Å, using TIP3P water model and the following other conditions: integration step of 2 fs coupling SHAKE algorithm; NPT ensemble at 1 bar pressure using the Berendsen barostat (Berendsen et al., 1998) with a time constant τp = 2.0 ps. The Particle mesh Ewald summation method (Darden et al., 1998) has been employed for the electrostatic potential long-range interactions with a 12 Å cutoff distance. In order to select different representative conformations of the system, root-mean square deviation (RMSD) based clustering of the whole trajectory has been performed using the agglomerative bottom-up approach available in Amber16 tools. After removing overall rotations and translations by RMS fitting the Cα atoms’ positions of the trajectory, the average linkage clustering algorithm has been applied, identifying 10 representative conformations of the protein. The complete MD analysis of the unbound M^pro^ has been reported in our previous study (Parise et al., 2021b).

The representative structure of the unbound Mpro, having the hydrogen on the N_δ_ of His41, and with the highest percentage of population was considered for the molecular docking approach performed by using AutoDock Vina (version 4.2) (Trott and Olson, 2010). The ligand and the SARS-CoV-2 M^pro^ models were processed using the AutoDock tools (ADT) to obtain the PDBQT (Protein Data Bank, Partial Charge (Q), and Atom Type (T)) coordinate files containing the information, namely polar hydrogen atoms, partial charges, correct atom types, and information on the articulation of flexible molecules. In particular, Gasteiger-Marsili charges were loaded in ADT. The substrate has been docked into a section of the crystal representing the minimal unit of ligand (37.5 × 15.2 × 37.6 Å) adopted in the simulations. The dimension box of 40 × 40 × 40 Å has been chosen to abundant cover the active site.

Before MD simulations of the M^pro^:H_4_Iso/M^pro^:H_3_Iso^−^ complexes, it was necessary to obtain H_4_Iso and H_3_Iso^−^ parameters. At this purpose gas-phase geometry optimization has been carried out using B3LYP/6-31G*. Atomic charges were derived by fitting the electrostatic potential according to the Merz–Singh–Kollman scheme, (Bayly et al., 1993), using the RESP procedure. Antechamber and parmchk modules of Amber16 (Case et al., 2017) have been employed to generate preparatory files to perform molecular mechanics (MM) relaxation of the complexes. 4 Na^+^ counter ions were added to neutralize the system for M^pro^:H_4_Iso and 5 Na^+^ for M^pro^:H_3_Iso^−^. The production step of 100 ns for the both complexes (M^pro^:H_4_Iso and M^pro^:H_3_Iso^−^) was performed using the same procedure of the apo-form simulation.

RMSD-based clustering of the entire trajectories was performed according to the relaxed complex scheme (RCS) protocol implemented in Amber 16 (Case et al., 2017) to provide a sampled and energetically accessible conformational ensemble. After removing the overall rotations and translations by RMS fitting of the positions of the Cα atoms of the trajectory, the average binding clustering algorithm implemented in cpptraj was applied to identify 10 clusters of representative conformations of the protein, described in Supplementary Table S4.

The resulting MD trajectories were used to assess the magnitude of structural changes in terms of root mean square deviation (RMSD), propensity of a given residue or region to shift, and root mean square fluctuation (RMSF).

The binding free energies between the ligand (H_4_Iso or H_3_Iso^−^) and M^pro^ were calculated by solving the linearised Poisson-Boltzman equation using the MM-PBSA (Molecular mechanics-Poisson Boltzman surface area) method, as implemented in the Amber code 16 (Case et al., 2017). The igb flag value of five associated with a salt concentration of 0.1 M was used. For the calculations, 100 frames of each MD trajectory over the last 50 ns were analysed.

## 3 Results and discussion

### 3.1 Chemical equilibria in water

**FIGURE 1:**
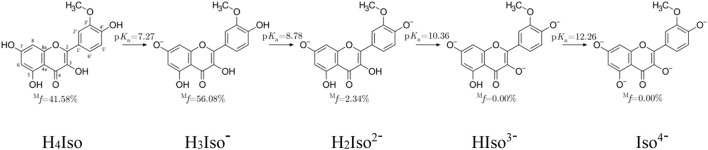
Dissociation constants and molar fractions for isorhamnetin species at pH = 7.4.

In understanding the chemical behavior of substances in aqueous solution, it is fundamental to know the relative acid-base equilibria. Sometimes the very low solubility in water of various antioxidants makes experimental measurements difficult. Modern computational chemistry offers a suitable alternative to such situations (Galano et al., 2016b). Figure 1 shows possibly present neutral and charged species of isorhamnetin in water solution, reported along with their p*K*_a_ values. The distribution diagram of isorhamnetin species as a function of pH is given in the supporting information section (Supplementary Figure S1).

The lowest p*K*_a_ value (7.27) was associated with the deprotonation of the–OH group located at the C_7_ position, followed by C_4'_ (8.78), C_3_ (10.36), and C_5_ (12.26). Also, for structurally similar scutellarein, chrysin and quercetin, deprotonation at the C_7_ position is preferred. Another important data useful for studying antioxidant properties in water is the molar distribution at physiological pH. Figure 1 evidences that the neutral (41.58%) and mono-anion (56.08%) species are found in the highest molar fractions. Contrary to these, the di-anion form is present at much lower, yet still non-negligible amount (2.34%). Therefore, these three species must be considered in the investigation.

### 3.2 Thermodynamic descriptors

Some indication on the occurrence of possible reaction pathways between radicals and antioxidant compounds can be obtained from adiabatic energy computations of certain molecular indicators, such as:

1) ionization energy (IP):

$$IP=∆H\left(Cx-{OH}^{.+}\right)+∆H\left(e^{-}\right)\;–∆H\left(Cx-OH\right)$$

2) proton affinity (PA):

$$PA=∆H\left(Cx-O^{-}\right)+∆H\;\left(H^{+}\right)\;–∆H\left(Cx-OH\right)$$

3) bond dissociation energy (BDE):

$$BDE=∆H\;\left(Cx-O∙\right)+∆H\left(H∙\right)\;–∆H\left(Cx-OH\right)$$

4) proton desorption energy (PDE):

$$PDE=∆H\left(Cx-O^{\cdot }\right)+∆H\;\left(H^{+}\right)\;–∆H\left(Cx-{OH}^{\cdot +}\right).$$

The obtained values are collected in Table 1.

**TABLE 1:** IP, BDE, PA, and PDE values calculated for neutral and charged isorhamnetin species in water and pentylethanoate solvents. All values are in kcal/mol.

Species	Solvent	OH position	IP	BDE	PA	PDE
H_4_Iso	Pentylethanoate	C_3_	131.3	73.8	61.0	9.2
		C_4'_		75.6	60.9	10.9
		C_5_		89.4	63.9	24.7
		C_7_		85.2	53.6	20.5
	Water	C_3_	118.4	75.8	32.6	7.2
		C_4'_		77.3	32.7	8.7
		C_5_		86.3	33.1	17.7
		C_7_		87.3	29.0	18.7
H_3_Iso^–^		C_3_	109.8	72.9	35.3	12.8
		C_4'_		75.9	33.8	15.8
		C_5_		84.7	39.1	24.7
H_2_Iso^2-^		C_3_	91.9	66.0	38.7	23.9
		C_5_		79.4	40.9	37.3

In the pentylethanoate solvent, commonly chosen to mimic a lipid-like environment, the BDE values indicate that the preferred site of dehydrogenation is the one involving the–OH group at the C_3_ position. The same behavior is observed for all the species present in water. The ionization potential in water decrease when moving from neutral to deprotonated forms, as do PA and PDE. Similar values and trends were also found in previous work done at the DFT level, but using a different exchange-correlation functional (B3LYP) (Thong et al., 2019).

### 3.3 Reactions in lipid-like and aqueous environments

The computed Gibbs free energies of reaction (ΔG) and activation (ΔG^‡^) for the considered mechanisms—HAT, SET, and RAF—of the reaction between the ∙OOH radical and isorhamnetin in the two considered environments are shown in Table 2. For RAF processes, we report only those with ΔG values less than 10 kcal/mol, since they are relevant from both thermodynamic and kinetic viewpoints.

**TABLE 2:** Gibbs free energies of reaction (ΔG) and activation (∆G^‡^) at 298.15 K in aqueous solution and pentylethanoate (indicated by^PE^ apex) for neutral and charged isorhamnetin species. All values are in kcal/mol.

Mechanism	H_4_Iso^PE^		H_4_Iso		H_3_Iso^–^		H_2_Iso^2-^	
	∆G	∆G^‡^	∆G	∆G^‡^	∆G	∆G^‡^	∆G	∆G^‡^
HAT-C_3_	−2.3	20.1	−3.3	18.6	−6.3	17.4	−13.1	−3.3
HAT-C_4'_	−0.5	21.3	−1.8	20.4	−3.2	19.7		
HAT-C_5_	13.2	—	7.2	28.6	5.6	36.4	0.3	27.2
HAT-C_7_	9.0	25.3	8.2	26.0				
RAF-OOH-C_2_	2.8	22.5	1.4	20.7	0.2	18.4	1.8	18.0
RAF-OOH-C_3_	9.5	20.8	10.2	—	6.7	18.4	5.9	14.1
RAF-OOH-C_3'_							8.7	31.6
SET	—	–	28.8	53.5	20.3	26.4	2.3	7.6

From Table 2, we see that the more favored thermodynamic process is the hydrogen atom transfer from the C_3_ position of neutral and charged isorhamnetin species to the ∙OOH radical in both lipid-like and aqueous environments. In particular, the latter cases provides a noteworthy observation on how the exergonic character of the process increases with the transition from neutral to mono- and di-anion forms. Also, the HAT processes from the C_4'_ position show negative Gibbs free energies, with a trend similar to HAT at the C_3_ site. The ∆G values for the mechanisms of radical addition and electron transfer reactions allows us to hypothesize that they can occur in both considered environments. Concerning the activation energies of the HAT-C_3_ and HAT-C_4'_ processes in water solvent, we observe that the corresponding values decrease with the increasing ionic character of the species.

**FIGURE 2:**
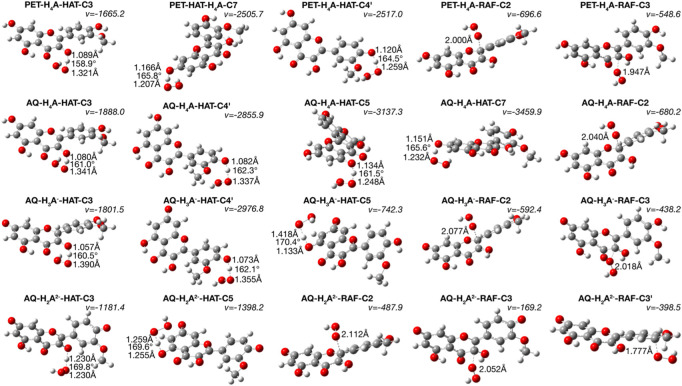
TSs structures for HAT and RAF mechanism. Distances are in Å and angles in degrees.

The rate constants and the branching ratios for the considered reaction mechanisms are reported in Table 3, while the transition state structures for HAT and RAF mechanisms are depicted in Figure 2.

**TABLE 3:** Rate constants (k)^a^ and branching ratios (*Γ*) of the reaction between isorhamnetin and *OOH radical in 1:1 ratio, computed at 298.15 K, for the different species present in solution at pH = 7.4

Mechanism	H_4_Iso^PE^		H_4_Iso		H_3_Iso^–^		H_2_Iso^2-^	
	k (M^−1^ s^−1^)	Γ(%)	k (M^−1^ s^−1^)	Γ(%)	k (M^−1^ s^−1^)	Γ(%)	k (M^−1^ s^−1^)	Γ(%)
HAT-C_3_	8.06 × 10^0^	31.9	1.48 × 10^2^	39.7	6.80 × 10^2^	50.3	2.49 × 10^9^	25.5
HAT-C_4'_	1.71 × 10^1^	67.6	2.52 × 10^2^	60.3	6.59 × 10^2^	48.7		
HAT-C_5_			9.34 × 10^−4^	0.0	5.76 × 10^−13^	0.0	2.22 × 10^−5^	0.0
HAT-C_7_	3.42 × 10^−3^	0.0	1.36 × 10^−2^	0.0				
RAF-C_2_	8.21 × 10^−3^	0.0	1.67 × 10^−1^	0.0	7.12 × 10^0^	0.0	1.24 × 10^1^	0.0
RAF-C_3_	1.18 × 10^−1^	0.5			6.03 × 10^0^	0.0	7.16 × 10^3^	0.0
RAF-C_3'_							1.23 × 10^−9^	0.0
SET	—	—	3.89 × 10^−27^	0.0	7.14 × 10^−9^	0.0	7.27 × 10^9^	74.5
Total	2.53 × 10^1^	3.73 × 10^2^	1.35 × 10^3^	9.76 × 10^9^				
Overall		1.55 × 10^2^	7.58 × 10^2^	2.28 × 10^8^				

^a^ The sum of the individual rate constants from the studied reaction routes was computed as “Total,” while “Overall” is the sum of the rate constants for the different species present in solution at pH = 7.4.

In the lipid-like phase, the total rate constant is 2.53 × 10^1^ and indicates that the process taking place mainly follows the HAT mechanism from the C_4'_-OH position. Due the presence of different charged and neutral species, on the other hand, the situation in water solution is different. In fact, for the H_4_Iso form the hydrogen abstraction process at the C_4'_ position is kinetically favored (k = 2.52 × 10^2^), followed by the one at the C_3_ site (1.48 × 10^2^). The total rate constant take into account the branching ratio that is 31.9% and 67.6% for C_3_ and C_4'_ site, respectively. In the mono-anion species, the antioxidant properties are essentially due to the SET mechanism as indicated by the k (8.20 × 10^9^) and the molar fraction values (74.5%). The same mechanism is favored by the H_3_Iso^–^form, but now, although the SET still gives the highest kinetic constant, the HAT mechanism from the C3 position also contributes considerably (Γ = 25.5%) to the total kinetic constant value. Considering the overall kinetic constant values, Table 3 clearly indicate that the isorhamnetin molecule has a high potential to scavenge ∙OOH radical (k = 4.60 × 10^9^), essentially owing to the mono- and di-anionic forms, underlining the importance of considering all species present at physiological pH.

Inspecting the TSs structures (Figure 2) of the attack of ∙OOH radical on one of the hydroxyl hydrogens provides reliable data on the breakage of the isorhamnetin-OH bonds and the formation of a new one with the radical, observed as the generation of H_2_O_2_ molecule. The presence of imaginary frequencies confirm this phenomenon.

A rationalization of the kinetic behaviors can be done also considering the electron spin densities of the radicals obtained after the abstraction of the hydrogens in the different positions. As shown in Supplementary Figure S2, the abstraction of a proton in C_3_ position induces an electron spin delocalization that involve the entire molecular structure, in both neutral and charged species and in both considered solvents, that stabilize the radicals. On the contrary, the hydrogen loss in position C_5_ gives a radical in which the electronic spin density is more concentrated in one side of the structure.

A comparison with some other antioxidants of similar structure, studied previously using very similar or identical computational protocols, is possible by examining the data reported in Table 4.

**TABLE 4:** K_overall_ values of Isorhamnetin and other structurally similar antioxidant against the OOH radical. Value are in M^−1^ s^−1^.

Molecules	*k*_overall_ (lipid-like)	*k*_overall_ (water)
Isorhamnetin	2.53 × 10^1^	4.60 × 10^9^
Scutellarin	3.57 × 10^−6^	7.09 × 10^5^
Scutellarein	1.06×10^3^	2.23 × 10^5^
Trolox	3.40 × 10^3^	8.96 × 10^4^
L1	1.89×10^4^	1.02 × 10^5^
Quercetin		8.11 × 10^9^
Daphnetin		1.51 × 10^7^

In pentylethanoate, the scavenging activity of isorhamnetin against the ∙OOH radical is relatively low and lower than that of the other compounds tested except for scutellarin (Spiegel et al., 2022b). On the contrary, in aqueous solution, its kinetic constant takes on a very high value (4.60 × 10^9^ M^−1^ s^−1^), comparable to that of quercetin (*k* = 8.11 × 10^9^ M^−1^ s^−1^) (Castaneda-Arriaga et al., 2020) and daphnetin (1.51 × 10^7^) (Boulebd and Khodja, 2021) and about five orders of magnitude higher than the corresponding value of Trolox (8.96 × 10^4^ M^−1^ s^−1^) (Alberto et al., 2013), which is generally used as a comparison to determine the antioxidant power of a molecule. On the other hand, isorhamnetin is a less efficient scavenger with respect to [4-(benzo[d]thiazol-2-yl)-2-((4,7-dimethyl-1,4,7-triazonan-1-yl)-methyl)-6-methoxyphenol] (L1 in Table 4) in the lipid-like phase, but more efficient in water solution (Spiegel et al., 2022a).

### 3.4 Copper chelating ability

The computations of the Gibbs energies (ΔG_f_) for the following reactions:

$$Cu{\left(H_{2}O\right)}_{4}^{2+}+H_{4}Iso\;\to \;{\left[Cu{\left(H_{2}O\right)}_{4}\times H_{4}Iso\right]}^{2+}\;;\;Cu{\left(H_{2}O\right)}_{4}^{2+}+2\;H_{4}Iso\;\to \;{\left[Cu{\left(H_{2}O\right)}_{4}\times 2\;H_{4}Iso\right]}^{2+}$$

$$Cu{\left(H_{2}O\right)}_{4}^{2+}+H_{3}{Iso}^{-}\;\to \;{\left[Cu{\left(H_{2}O\right)}_{4}\times H_{3}{Iso}^{-}\right]}^{+}\;;\;Cu{\left(H_{2}O\right)}_{4}^{2+}+2\;H_{3}{Iso}^{-}\;\to \;\left[Cu{\left(H_{2}O\right)}_{4}\times 2\;H_{3}{Iso}^{-}\right]$$

$$Cu{\left(H_{2}O\right)}_{4}^{2+}+H_{2}{Iso}^{2-}\;\to \;\left[Cu{\left(H_{2}O\right)}_{4}\times H_{2}{Iso}^{2-}\right];\;Cu{\left(H_{2}O\right)}_{4}^{2+}+2\;H_{2}{Iso}^{2-}\;\to \;{\left[Cu{\left(H_{2}O\right)}_{4}\times 2H_{2}{Iso}^{2-}\right]}^{2-}$$

made it possible to establish the Cu^2+^chelating power of isorhamnetin. The relative apparent equilibrium constants (K^app^) were calculated using the following expressions (Perez-Gonzalez et al., 2020):

$$K^{app}=\sum K_{i}^{II}$$

KiII=∑Kf*fm

where $K_{i}^{II}$ equals $\sum K_{f}$ multiplied by the molar fraction, fm, of the species under consideration at pH 7.4; $\sum K_{f}$ is the sum of $K_{f}$ for all possible complexation sites; and $K_{f}$ ( $K_{f}$ = e ^−∆Gf/RT^) represents each reaction pathway that contributes to the chelation process.

**FIGURE 3:**
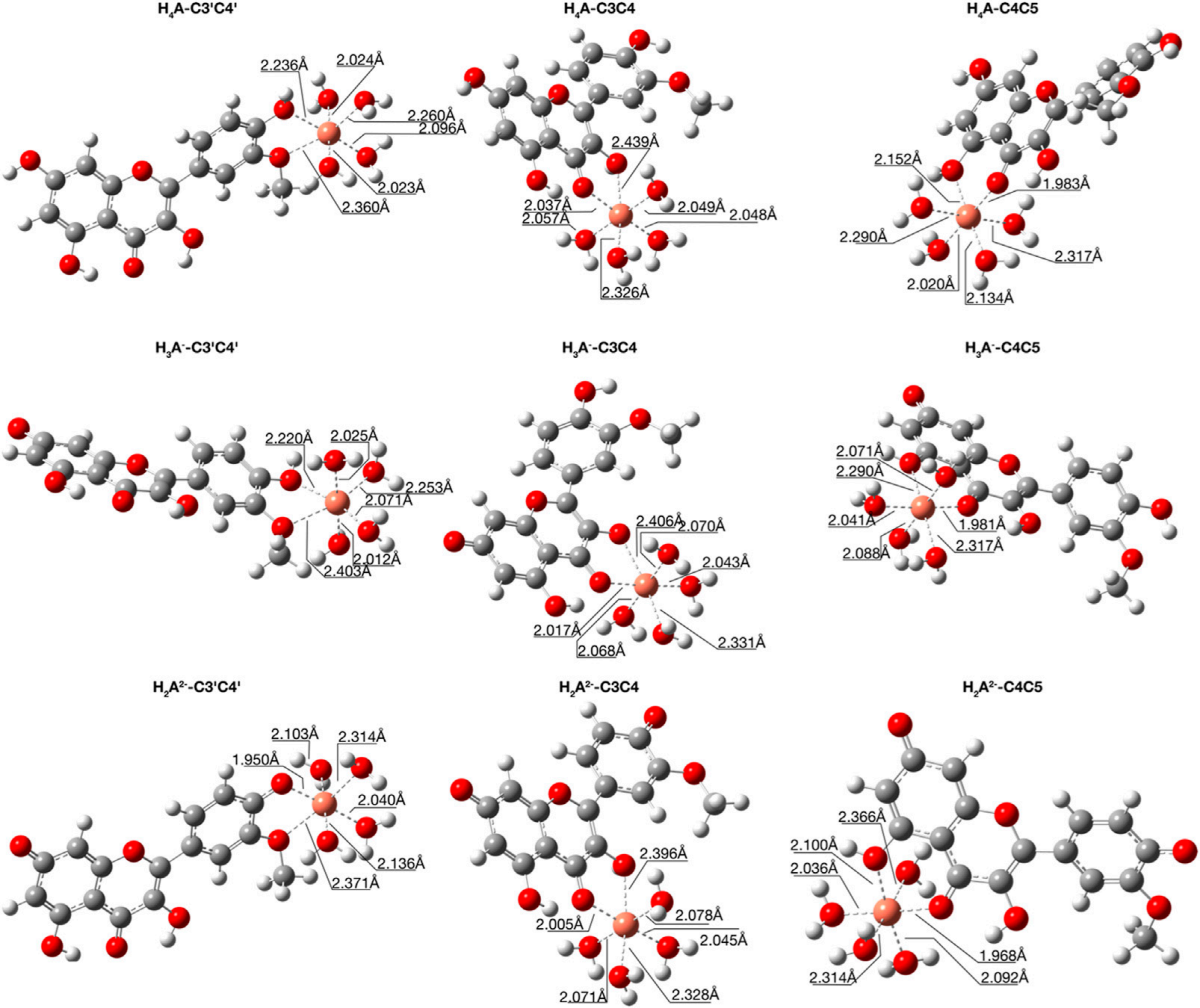
Structures for the most stable 1:1 complexes between Cu^2+^ and the neutral and charged species of isorhamnetin in water solvent.

We considered three different chelating sites, which include oxygens at the C_3'_C_4'_, C_3_C_4_ and C_4_C_5_ positions. The results for a 1:1 M ratio are reported in Table 5, and the related structures are displayed in Figure 3. For neutral and mono-anionic isorhamnetin, the most stable complex is the one with copper being coordinated at the C_3_-C_4_ site, while for H_2_Iso^2–^ the preferred one is C_3'_C_4'._ In all cases, the thermodynamic stability of the complexes increases with the ionic character of the ligand.

**TABLE 5:** Gibbs formation energies (ΔG_f_), their differences (ΔΔG_f_), both in kcal/mol, and kinetic constants ( $K_{f}$ , $\sum K_{f}$ , $K_{f}^{II}$ , and $K_{i}^{app}$ in M^−1^ s^−1^) for the different coordination sites of isorhamnetin with Cu(II) ion in 1: 1 ratio.

Coordination site	ΔG_f_	ΔΔGf	$K_{f}$	$\sum K_{f}$	$K_{f}^{II}$
H_4_Iso (41.58%)					
C_3'_C_4'_	1.7		5.87 × 10^−2^	9.57 × 10^2^	3.98 × 10°
C_3_C_4_	−1.3		9.47×*x*10°		
C_4_C_5_	1.8		4.88 × 10^−2^		
H_3_Iso^–^ (56.08%)					
C_3'_C_4'_	0.7		2.83 × 10^−1^	1.79 × 10^2^	1.00 × 10^2^
C_3_C_4_	−3.1		1.76×10^2^		
C_4_C_5_	−0.4		2.01×10°		
H_2_Iso^2−^ (2.34%)					
C_3'_C_4'_	−16.7		1.95×10^12^	1.95 × 10^12^	4.56 × 10^10^
C_3_C_4_	−4.7		2.98×10^3^		
C_4_C_5_	−4.2		1.21×10^3^		
$K_{f}^{app}$ = 4.56×10^10^					

The computed $\sum K_{f}$ reflects the obtained ΔG_f_, with that for Iso^2–^ being the greatest among all the considered species. Taking into account the molar fraction of the ligand at physiological pH, the apparent equilibrium constant becomes equal 4.56 × 10^10^ M^−1^ s^−1^, implying that all species present in the water solvent equilibrium must be taken into account to obtain a reliable outcomes. Comparison with other systems treated at the same level of theory reveals that the copper chelating power of isorhamnetin is lower than that of scutellarin and scutellarein, as the $K_{f}^{app}$ values of these systems are 4.77 × 10^20^ and 1.29 × 10^12^, respectively (Spiegel et al., 2022b).

From Figure 3, where the optimized structures and main geometrical parameters are reported, we note the distorted tetrahedral topology around the Cu^2+^ ion, which is coordinated with two ligand oxygens and two water molecules with Cu-O distances that range from about 1.9 Å to 2.1 Å. The other two water molecules act as a micro-solvation sphere.

**FIGURE 4:**
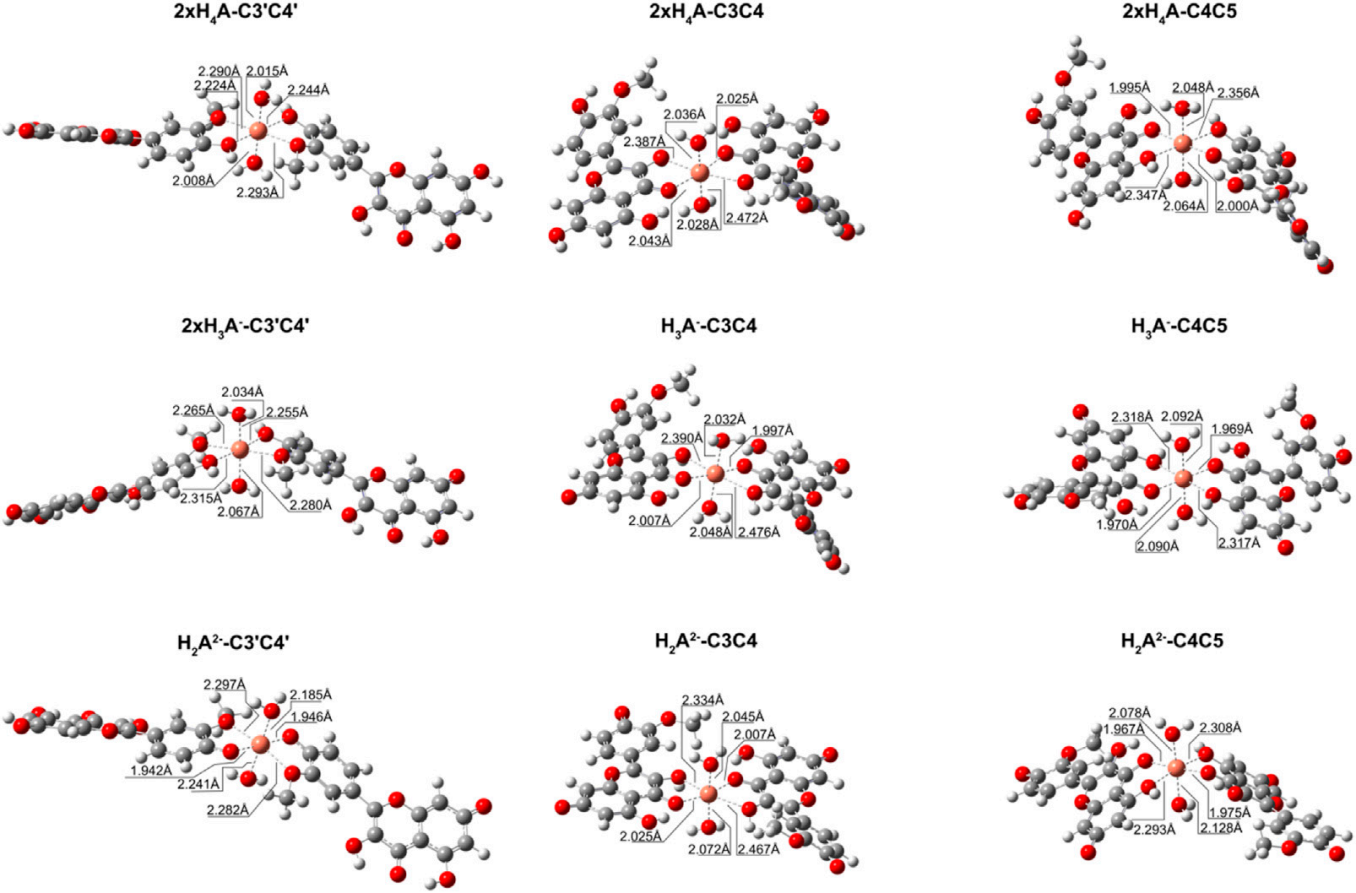
Structures for the most stable 1:2 complexes between Cu^2+^ and the neutral and charged species of isorhamnetin.

We also explored what happens when the Cu^2+^ ion interacts with isorhamnetin at molar ratios of 1: 2. The results are summarized in Table 6. For all considered ligand species, the preferred coordination site is C_3_C_4_, and the most stable complex is the one in which the copper cation is coordinated by two H_2_A^2−^ forms (ΔG_f_ = −14.9 kcal/mol). From Figure 4, it can be proven that the structural topologies are different: the coordination with two charged ligands results in a butterfly-like structure, with the coordinated antioxidant oxygens distances that are different. As in the case of the 1:1 ratio, three coordinate H_2_O strongly interact with the copper center.

**TABLE 6:** The Gibbs formation energies (ΔG_f_), their differences (ΔΔG_f_), both in kcal/mol, and kinetic constants ( $K_{f}$ , $\sum K_{f}$ , $K_{f}^{II}$ and $K_{i}^{app}$ in M^−1^ s^−1^) for the different coordination sites of isorhamnetin with Cu (II) ion in 1:2 ratio.

Coordination site	ΔG_f_	ΔΔGf	$K_{f}$	$\sum K_{f}$	$K_{f}^{II}$
H_4_Iso (41,58%)					
C_3'_C_4'_	1.5		8.36 × 10^−2^	2.12 × 10^5^	8.82 × 10^4^
C_3_C_4_	−7.3		2.12 × 10^5^		
C_4_C_5_	−2.1		3.54 × 10^4^		
H_3_Iso^–^ (56.08%)					
C_3'_C_4'_					
C_3_C_4_	−12.2		9.64 × 10^8^		
C_4_C_5_	−9.7		1.38 × 10^7^		
H_2_Iso^2−^(2.34%)					
C_3'_C_4'_				1.73 × 10^25^	4.06 × 10^23^
C_3_C_4_	−14.9		8.96 × 10^10^		
C_4_C_5_	−9.3		7.23 × 10^6^		
$K_{f}^{app}$ = 4.06x10^23^					

Comparing the $K_{f}^{app}$ value obtained for the 2:1 chelates with that computed for those at a 1:1 M ratio, it can be seen that the formers are associated with higher complexation power. Given previous studies that considered a 1:1 complexes of antioxidants with copper, we note that the chelating power of Iso ( $K_{f}^{app}$ = 4.56 × 10^10^) is lower than that of scutellarin and scutellarein, which show $K_{f}^{app}$ values of 4.77−10^20^ and 1.29 × 10^12^ M^−1^ s^−1^, respectively (Thong et al., 2019).

In order to explore the spectroscopic changes upon Cu^2+^ complexation, we calculated the excitation energies for both the isolated isorhamnetin and its complexes with copper (see Supplementary Table S1). The lowest energy transition (S_1_) for the bare molecule, characterized by high oscillator strength and being of HOMO- > LUMO in nature (more than 90%), undergo a sensible bathochromic shift starting from H_4_Iso (325.4 nm), to H_3_Iso^−^ (343.2 nm) and H_2_Iso^2−^ (373.6 nm). After Cu^2+^ complexation, the peak that undergoes the major shift at higher wavelengths is related to the C_5_C_6_ coordination site in all the species and different molar ratios. In particular, the highest wavelength (410 nm) is observed for the C_5_C_6_ coordination site with the isorhamnetin mono-anion in the 1:1 metal-ligand molar ratio. The excited energies in the complexes continue to be characterized by HOMO- > LUMO transitions.

### 3.5 Reduction mechanism with O_2_^•−^ and Asc^−^

The pro-oxidant activity can be theoretically evaluated by studying the Cu(II)- > Cu(I) reduction reaction of the given metal-antioxidant complex (Fenton’s reaction) that leads to the production of *OH radicals. For this purpose, the reduction reaction of the studied complexes (in molar ratios of 1:1 and 1:2) with the reducing agents present in the physiological environment, such as superoxide (O_2_^−^) and ascorbic acid (Asc^−^), were studied. Thermodynamic and kinetic results are reported in Table 7 (1:1 M ratio complexes) and Supplementary Table S2 (1:2 M ratio complexes), along with the results of an analogous reactions but with a solvated copper ion only, which were taken as a reference.

**TABLE 7:** The standard enthalpy (Δ_r_H°, kcal/mol), Gibbs free energy of reaction (Δ_r_G°, kcal/mol), reorganization energy (λ, kcal/mol), Gibbs free energy of activation (Δ_r_G^‡^, kcal/mol), diffusion rate constant (k_D_, M^−1^ s^−1^), TST thermal rate constant (k_T_, M^−1^ s^−1^), diffusion-corrected apparent rate constant (k_app_, M^−1^ s^−1^) calculated at 298.15 K for the redox reaction between the copper complexes and two reducing agents (O_2_^•−^ and Asc^−^) in water.

Species	Position	Δ_r_H°	Δ_r_G°	λ	Δ_r_G^‡^	K_D_	k_app_
*[isorhamnetin • Cu*(*H*_*2*_ *O)*_*2*_ *]* ^*2+*^ *+ O*_*2*_^*•*^ *→[isorhamnetin • Cu*(*H*_*2*_ *O)*_*2*_ *]* ^*+*^ *+ O*_*2*_							
		41.9	36.5	34.1	36.5	7.70 × 10^9^	1.01 × 10^−14^
H_4_Iso	C_3'_C_4'_	31.5	29.9	34.2	30.0	8.31 × 10^9^	6.02 × 10^−10^
	C_3_C_4_	37.6	35.4	35.0	35.4	8.35 × 10^9^	6.93 × 10^−14^
	C_4_C_5_	37.4	34.3	32.4	34.4	8.37 × 10^9^	4.27 × 10^−13^
H_3_Iso^−^	C_3′_C_4′_	32.3	30.0	35.4	30.2	8.39 × 10^9^	4.47 × 10^−10^
	C_3_C_4_	38.5	34.8	37.3	34.9	8.37 × 10^9^	1.77 × 10^−13^
	C_4_C_5_	39.6	35.5	37.8	35.5	8.26 × 10^9^	5.54 × 10^−14^
H_2_Iso^2−^	C_3′_C_4′_	42.3	36.9	41.3	37.0	8.41 × 10^9^	4.54 × 10^−15^
	C_3_C_4_	40.4	35.9	37.6	36.0	8.36 × 10^9^	2.89 × 10^−14^
	C_4_C_5_	37.8	39.1	32.6	39.5	8.56 × 10^9^	7.80 × 10^−17^
		29.3	23.0	29.7	23.41	7.43 × 10^9^	4.45 × 10^−5^
H_4_Iso	C_3′_C_4′_	18.9	16.4	29.8	17.9	7.50 × 10^9^	4.64 × 10^−1^
	C_3_C_4_	25.0	21.9	30.6	22.5	7.52 × 10^9^	1.93 × 10^−4^
	C_4_C_5_	24.9	20.9	28.1	21.3	7.52 × 10^9^	1.36 × 10^−3^
H_3_Iso^−^	C_3′_C_4′_	19.7	16.6	31.0	18.2	7.53 × 10^9^	2.58 × 10^−1^
	C_3_C_4_	25.9	21.4	33.0	22.4	7.52 × 10^9^	2.34 × 10^−4^
	C_4_C_5_	27.0	22.0	33.4	23.0	7.49 × 10^9^	8.75 × 10^−5^
H_2_Iso^2−^	C_3′_C_4′_	19.7	16.6	43.8	20.8	7.53 × 10^9^	3.38 × 10^−3^
	C_3_C_4_	25.9	21.4	34.3	22.6	7.52 × 10^9^	1.65 × 10^−4^
	C_4_C_5_	27.0	22.0	31.9	22.8	7.59 × 10^9^	1.27 × 10^−4^

In all the studied systems, the reaction with the superoxide is more exergonic than the corresponding one with the ascorbic acid anion, as also noted previously for the pyridoxal antioxidant (Ngo et al., 2022).

Regarding the reaction with O_2_^−^, we found its feasibility to be greatest among neutral and mono-anionic forms of isorhamnetin with the C_3’_C_4'_, and then the C_4_C_5_ coordination sites occupied. The relative kinetic constants indicate that the reduction reaction of these kind of complexes favors the reduction of the copper ion to a lower oxidation state.

Turning to the study of what happens when the Asc anion is used to reduce the copper ion in the Fenton’s reaction, we first note that the Gibbs reaction energies are much less exothermic than for the reactions with the superoxide ion. Indeed, they fall in the range of 16.4–22.0 kcal/mol for the complexes with molar ratio 1:1 and 15.3–29.0 kcal/mol (excluding the value of 53.8 kcal/mol found for the C_3'_C_4'_ coordination site) for the complexes with a stoichiometry of 1:2.

From the values of the kinetic constants, it can be seen that the formation of complexes promotes the process of copper reduction towards lower oxidation states. In general, we can support the fact that the presence of ascorbic acid causes a greater pro-oxidant hazard than the reaction with the superoxide radical does, as previously noted for other antioxidants (Ngo et al., 2022).

### 3.6 Inhibitory activity

Flavonoids have been proven to be effective inhibitors of several enzymes involved in several biological and medical processes. In particular, isorhamnetin has been proposed as a promising inhibitor of the cyclooxygenase-2 (Seo et al., 2014), lactate dehydrogenase, adenosine diphosphate and other enzymes. Recently, some flavonoids have been proposed as non-covalent inhibitors of the main protease (M^pro^) protein which plays an important role in SARS-CoV-2 main protease enzyme infection (Abian et al., 2020; Puttaswamy et al., 2020; Rizzuti et al., 2021).

M^pro^ explains its action in the cleavage of polyproteins at many sites generating non-structural proteins, relevant in the replication process of the virus (Anand et al., 2003), such as endo- and exo-ribonuclease as well as RNA polymerase. So, it is an important target for the development of new anticoronavirus therapeutic agents (Yang et al., 2005; Pillaiyar et al., 2016). From a structural point of view, SARS-CoV-2 M^pro^ is an homodimer and each protomer is characterized by three domains connected by a loop region. The catalytic center contain two crucial residues (His41 and Cys145) and catalyze the cleaving the of the polyprotein, translated from the viral RNA at different positions, generating proteins that contribute in the arresting process of the viral replication cycle (Ullrich and Nitsche, 2020).

Since the global health emergency generated by coronavirus disease 2019 is still in progress, we considered interesting to verify whether the isorhamnetin, coming from plants spread in various continents, can be a substrate capable to inhibit the M^pro^.

**FIGURE 5:**
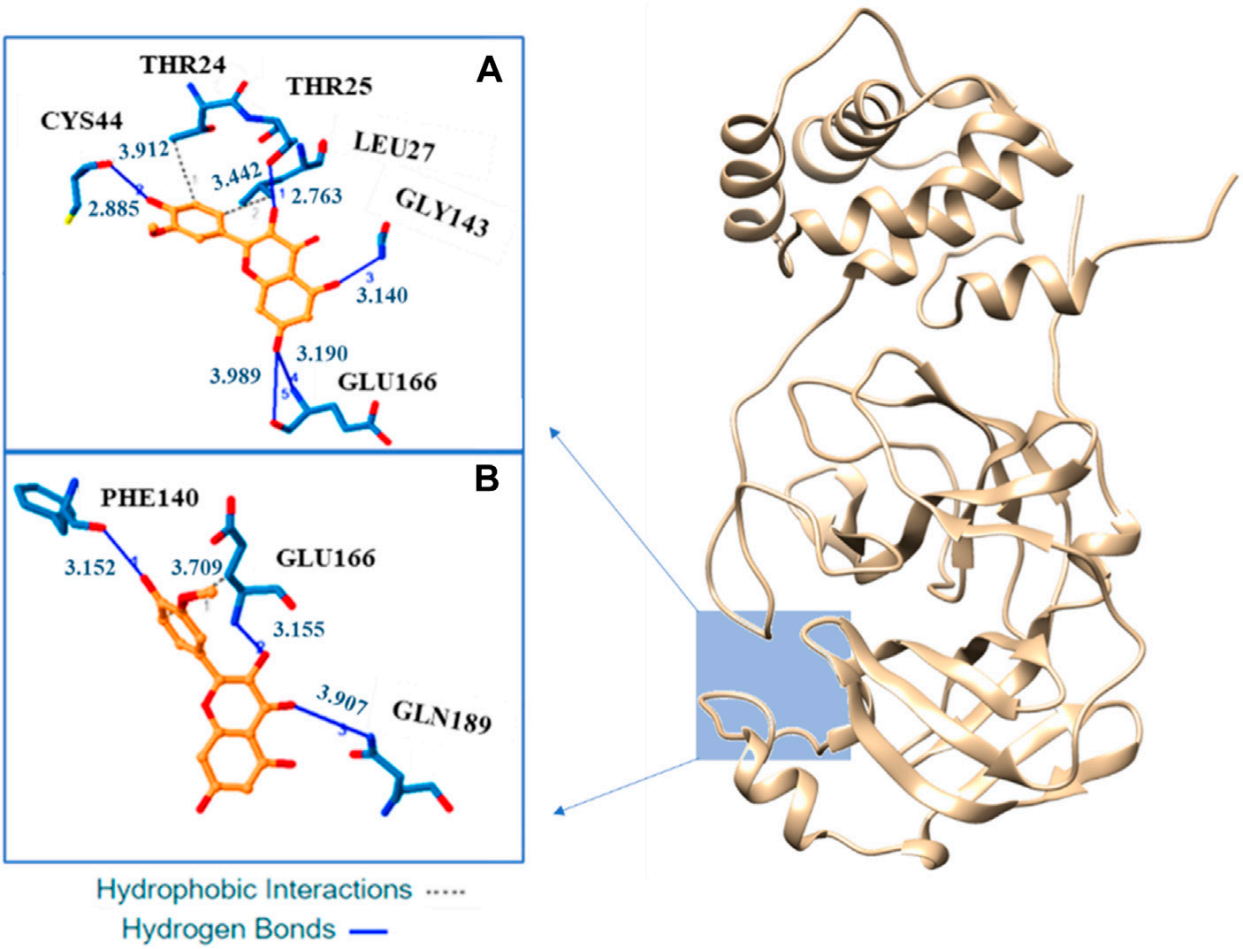
Three-dimensional representation of the best docking pose for H_4_Iso **(A)** and H_3_Is^−^ **(B)** species. The residues that have crucial contacts with the compounds are shown in the square windows **(A,B)**, for H_4_Iso and H_3_Is respectively.

Our docking analysis shows as isorhamnetin and its mono-anionic form establish many hydrophobic interactions (HI) and hydrogen bonds (H-bond) with different aminoacids in the enzyme catalityc pocket (see Figure 5 and Supplementary Table S3). For H_4_Iso two HI are established with the Thr24 and Leu27 while for H_3_Iso^−^ only the interaction with Glu166 is present. Five H-bonds, with Cys44, Thr25, Gly143, and Glu166, are present in the binding of isorhamnetin with M^pro^. In the case of H_3_Iso^−^ they involve the Phe140, Glu166, and Gln189. In both the systems their lenhgts range from 2.91 to 3.99 Å (see Figure 5).

Albeit with different amino acid residues, the binding energy values for both the neutral and anionic forms (Supplementary Table S3) result to be very close. The found value for the best pose (−6.1 kcal/mol) well agrees with that proposed for quercetin thoughout isothermal titration calorimetry that result to be −7.6 kcal/mol (Abian et al., 2020) and with the computed binding energy between eugenol and Mpro (Rizzuti et al., 2021). Furthermore, our computed binding energy is sligtly higher than that obtained for Ginkgetin (−9.5 kcal/mol), Delphinidin (−9.4 kcal/mol), Cyanidin 3,5-diglucoside, (−9.4 kcal/mol) and Amentofavone (−9.7 kcal/mol) antioxidants with similar structures (Puttaswamy et al., 2020). Finally, a comparison can be made also with the binding energies of quercetin and its anion with furin protein that result to be −7.8 and −7.7 kcal/mol, respectively (Milanovic et al., 2021).

The molecular docking examination induced us to perform MD silmulations with the aim to observe the dynamic behaviour of the complexes Mpro:H_4_Iso and Mpro:H_3_Iso. The analysis of their MD trajectories indicated a different behaviour of the two tested molecules, despite starting from the docked pose where both the ligands were located in proximity of catalytic site. During the simulation time in correspondence of ∼20 ns, the anionic form goes to a distal area from the catalytic pocket generating a different RMSD trend (Supplementary Figure S3) from that obtained in the case of Mpro:H4Iso. In particular, the H_3_Iso-is placed in a site defined by Ile213, Pro252-Leu253, Gln256, Val296-Val297, Cys300-Gly302 residues. The Supplementary Figure S4 related to RMSF value evidenced a different fluctuation in the related region. This site corresponds to the named site #3, one of the six allosteric sites experimentally proposed (Douangamath et al., 2020; El BabaLutomski et al., 2020; Günther et al., 2021) and then *in silico* observed (Alzyoud et al., 2022).

**FIGURE 6:**
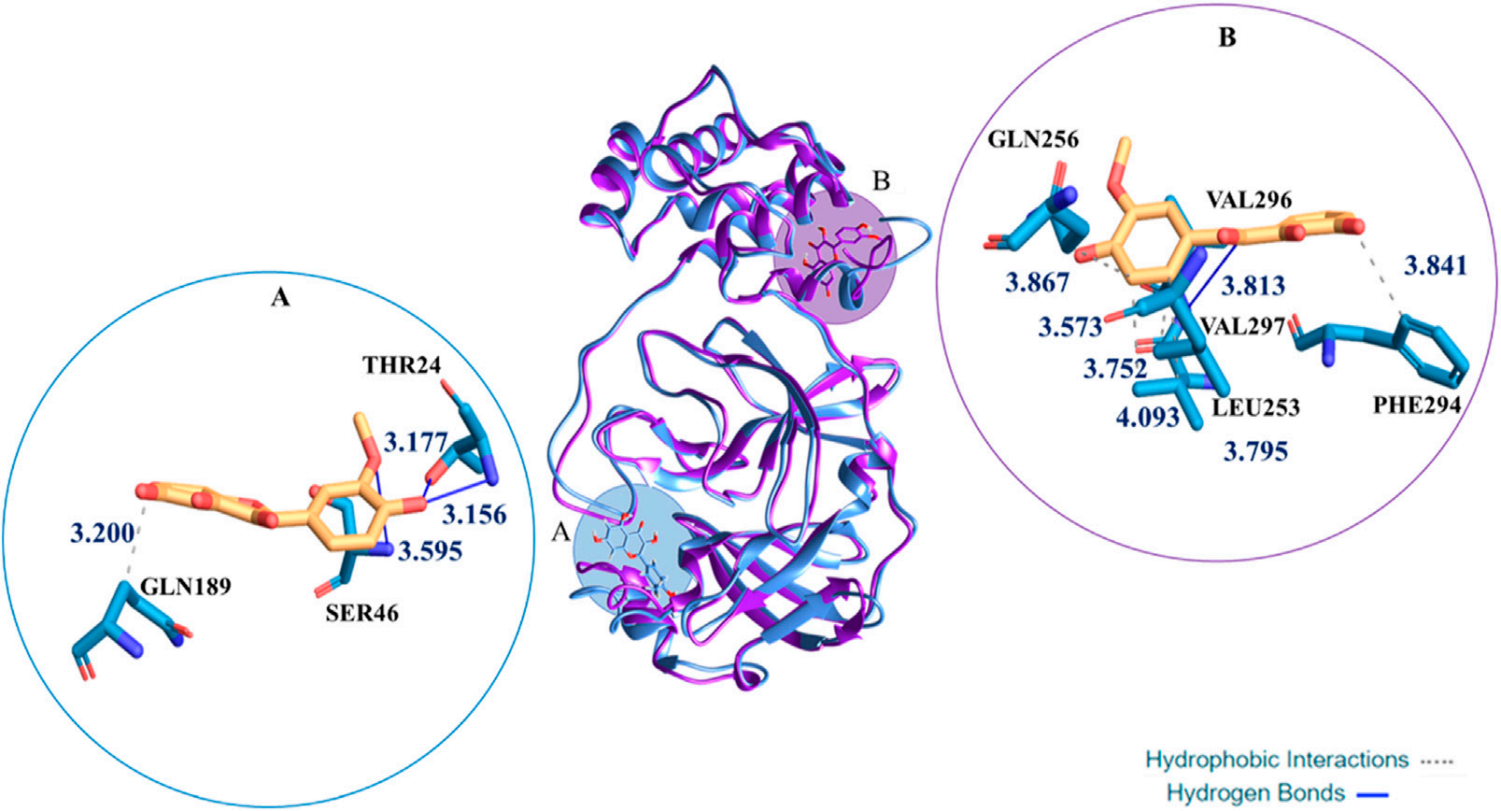
Superimposition of the most representative cluster from 100 ns of MD for the M^pro^:H_4_Iso and M^pro^:H_3_Iso^−^. In round windows complexes three-dimensional representation of the clustered structures with the residues that have crucial contacts with the compounds, in **(A)** (catalytic site) the H_4_Iso, in **(B)** (site #3) the H_3_Iso^−^ one.

In fact, the M^pro^ enzyme includes several pockets on its surface believed important for its catalytic activity; some of them exist in distal areas from the main catalytic pocket. The site #3 located at the dimer interface showed poor druggability due to its very small and shallow cavity that is significantly less hydrophilic. The protease movement during the MD of M^pro^:H_3_Iso^−^ makes more exposed such distal sites, making them more accessible. This does not take place during the MD of M^pro^:H_4_Iso as it can be evinced from a Figure 6 where the superposition of the most representative structure of the complexes Mpro:H_4_Iso and Mpro:H_3_Iso-is reported. Furthermore the ligand affinity to the M^pro^, evaluated in terms of ΔG binding, calculated *via* MMPBSA method proposed also the neutral form, H_4_Iso, as the most thermodynamically favorable. (see Supplementary Table S5) This outcome better clarifies the different nature of the interactions generated by the two ligands in the two different sites.

The possible coexistence of the two forms (neutral and anionic), at physiological pH, could be helpful to enhance the antiviral response of the isorhamnetin molecule providing provide a good (natural) starting point for lead optimization chemistry to disactivate the SARS CoV-2 M^pro^.

## 4 Conclusion

In the present work, we have shown, by examining the isorhamnetin molecule, how quantum mechanics methods based on density functional theory can provide useful and reliable information on geometric and electronic structures, chemical equilibria in solution, and reaction mechanisms of antioxidant systems. The results show that.

• isorhamnetin in aqueous solution and at physiological pH (7.4) exists in neutral, mono- and di-anionic forms (molar fractions equal 41.58%, 56.08%, and 2.34%, respectively) rendering them important to consider in calculations aiming to provide reliable kinetics of the reactivity towards *OOH radical;
• it is important to consider different molar ratios (1:1, 1:2) to determine the chelating capacity towards copper ions;
• the reduction process of the obtained complexes with the reducing agents present in the physiological environment (superoxide anion and absorbic acid anion) are essential for predicting their chemical behavior in Fenton’s reactions;
• isorhamnetin has good non-covalent inhibitory potency towards M^pro^, which is a pharmacological target for the treatment of SARS-CoV-2 infection.

## Data Availability

Publicly available datasets were analyzed in this study. This data can be found here: https://www.rcsb.org/- PDB code 6W63.
