# Supplementary material for: Computational investigation on the antioxidant activities and on the Mpro SARS-CoV-2 non-covalent inhibition of isorhamnetin
Source: Front Chem. 2023 Jan 24;11:1122880. doi: 10.3389/fchem.2023.1122880 (PMC9902383; doi:10.3389/fchem.2023.1122880)
Supplement: Supplementary file 1 [file DataSheet1.pdf]

Supplementary Material

- **Computational Investigation on the Antioxidant Activities and on the M^{pro} SARS-CoV-2 non-covalent inhibition of Isorhamnetin**

Maciej Spiegel[1,2], Giada Ciardullo[1], Tiziana Marino[1], Nino Russo[1,*]

[1] Dipartimento di Chimica e Tecnologie Chimiche, Università della Calabria, I-87136 Rende (CS), Italy

[2] Department of Pharmacognosy and Herbal Medicines, Wroclaw Medical University, Borowska 211, 50-556 Wroclaw, Poland

***Correspondence:**
Nino Russo
nino.russo@unical.it

Supplementary Data

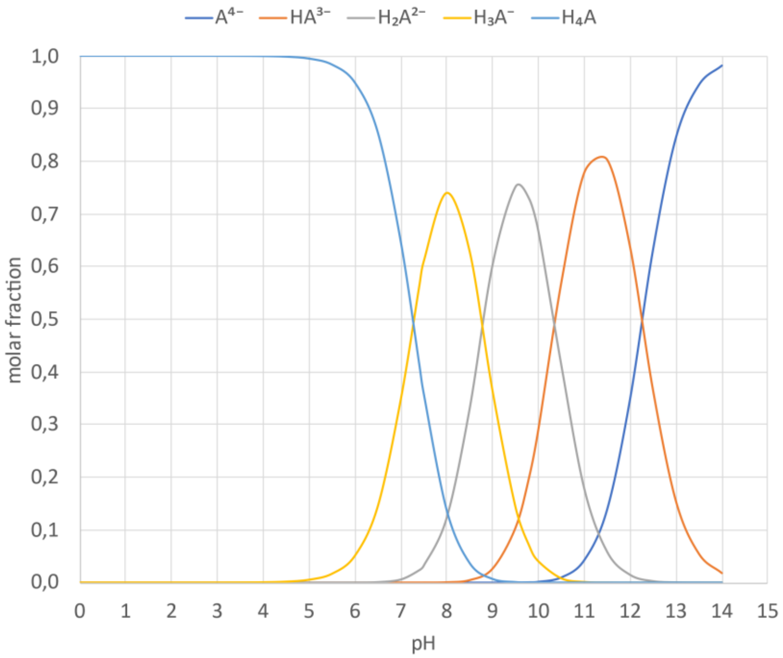

0	1–	2-	3–	4–

H_4A (41.58%)	H_3A^- (56.08%)	H_2A^{2-} (2.34%)	HA^{3-} (0.00%)	A^{4-} (0.00%)

Figure S1. Chemical equilibria of isorhamnetin species in water and molar fractions at pH=7.4

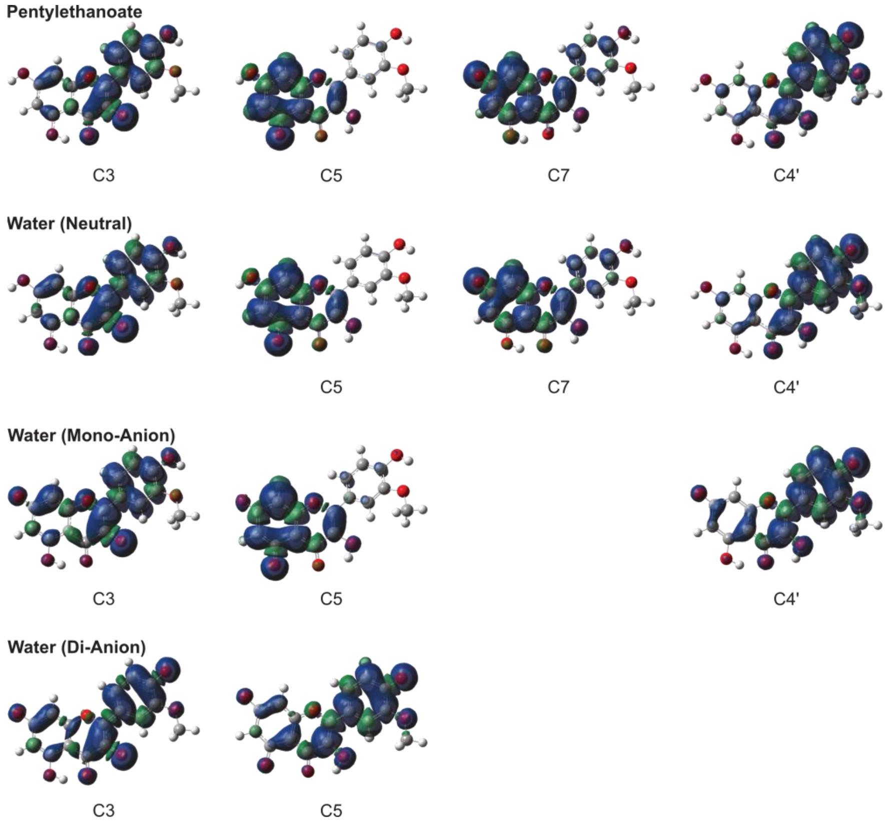

Figure S2. Electron spin densities for neutral and charged Isorhamnetin in pentylethanoate and water solvents.

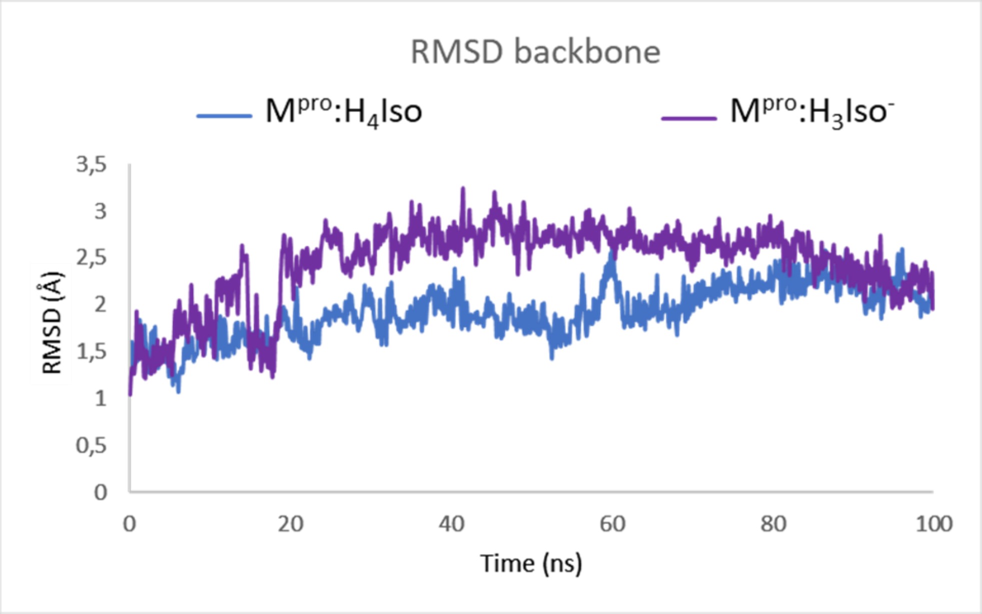

Figure S3. RMSD plot calculated for backbone atoms of the M^{pro} complexes

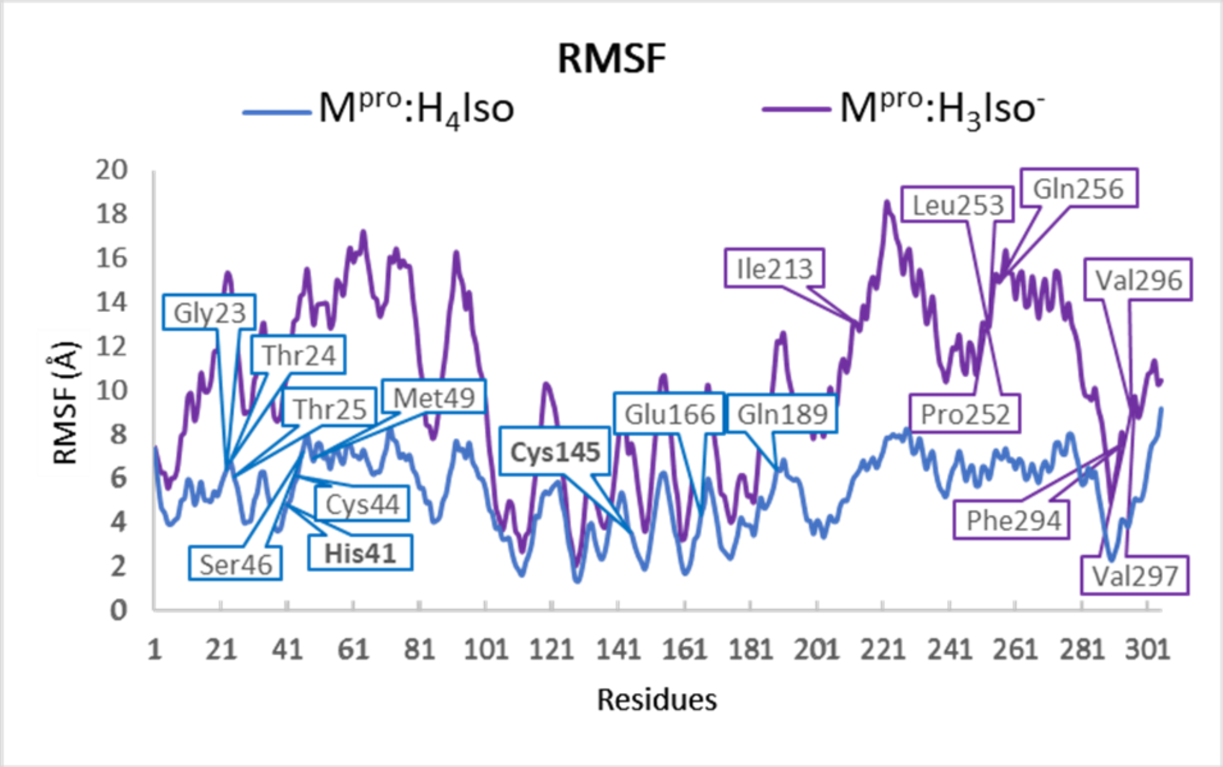

Figure S4. RMSF plots calculated for the M^{pro} complexes.

Table S1. Vertical excitation energy, ΔE (eV), λ (nm), oscillator strength *f*, and main transitions for the isorhamnetin neutral and charged species (S_1) and their 1:1 and 1:2 molar ratio Cu^{2+} complexes (coordination sites $C_{3'}C_{4'}$, C_4C_5, C_5C_6) in water using M052X/6-311+g(d,p).

**Complexation Site**	**State**	**λ**	**ΔE**	***f***	**Main Transition**
H_4Iso					
S_1		325.4	3.81	0.701	H -> L (91.7%)
$C_{3'}C_{4'}$		322.1	3.85	0.674	
C_4C_5		330.0	3.76	0.693	
C_5C_6		346.4	3.58	0.787	
H_3Iso^-					
S_1		343.2	3.61	0.603	H -> L (94.3%)
$C_{3'}C_{4'}$		316.2	3.92	0.151	
C_4C_5		353.8	3.50	0.573	

C_5C_6	359.0	3.45	0.626	
H_2Iso^{2-}				
S_1	373.6	3.32	0.909	H -> L (92.3%)
$C_{3'}C_{4'}$	360.7	3.44	0.866	
C_4C_5	334.6	3.71	0.224	
C_5C_6	410.0	3.02	0.250	
**1:2 molar ratio**	**λ**	**ΔE**		***f***
H_4Iso				
$C_{3'}C_{4'}$	322.0	3.85		1.045
C_4C_5	331.1	3.74		1.053
C_5C_6	344.1	3.60		1.476
H_3Iso^-				
$C_{3'}C_{4'}$	345.2	3.592		1.056
C_4C_5	354.3	3.500		1.086
C_5C_6	359.0	3.454		0.650
H_2Iso^{2-}				
$C_{3'}C_{4'}$	358.5	3.458		1.681

C_4C_5	389.7	3.182	1.481
C_5C_6	405.2	3.060	1.694

Table S2**.** The Gibbs free energy of reaction ($\Delta_rG°$, kcal/mol), reorganization energy (λ, kcal/mol), Gibbs free energy of activation ($\Delta_rG^{‡}$, kcal/mol), diffusion-corrected apparent rate constant (k_{app}, M^{-1} s^{-1}) calculated at 298.15K for the redox reaction between the copper-isorhamnetin (1:2) complexes and two reducing agents ($O_2^{\bullet -}$ and Asc^-) in water.

**Species**	**Position**	**$\Delta_rG°$**	**λ**	**$\Delta_rG^{‡}$**	**k_{app}**
[2 isorhamnetin • $Cu(H_2O)_2]^{2+}$ + $O_2^{\bullet -}$ →[2 isorhamnetin • $Cu(H_2O)_2]^{+}$ + O_2					
	C3’C4’	27.9	29.7	27.9	$2{,}09\times10^{-8}$
**H_4Iso**	C3C4	34.6	35.2	34.6	$2{,}67\times10^{-13}$
	C4C5	36.8	33.1	36.9	$5{,}50\times10^{-15}$
	C3’C4’	66,2	24,8	83,5	$3{,}96\times10^{-49}$
**H_3Iso^{-}**	C3C4	37,3	37,3	37,3	$2{,}81\times10^{-15}$
	C4C5	40,8	33,8	41,2	$4{,}15\times10^{-18}$
	C3’C4’	41,4	42,9	41,4	$2{,}72\times10^{-18}$
**H_2Iso^{2-}**	C3C4	39,0	36,0	39,1	$1{,}44\times10^{-16}$
	C4C5	38,8	37,5	38,8	$2{,}20\times10^{-16}$

[2 isorhamnetin • Cu(H_2O)$_2$]$^{2+}$ + Asc$^-$ →[2 isorhamnetin • Cu(H_2O)$_2$]$^{+}$ + Asc$^{\bullet}$

	C3’C4’	15,5	24,3	16,3	$7{,}02\times10^{0}$
**H_4Iso**	C3C4	22,2	29,8	22,7	$1{,}46\times10^{-4}$
	C4C5	24,4	27,7	24,5	$6{,}82\times10^{-6}$
	C3’C4’	53,8	19,4	69,1	$1{,}49\times10^{-38}$
**H_3Iso^{-}**	C3C4	25,0	31,8	25,36	$1{,}58\times10^{-6}$
	C4C5	28,4	28,4	28,4	$9{,}41\times10^{-9}$
	C3’C4’	29,0	35,5	28,4	$9{,}48\times10^{-9}$
**H_2Iso^{2-}**	C3C4	26,6	30,6	26,7	$1{,}58\times10^{-7}$
	C4C5	26,4	32,0	26,7	$1{,}82\times10^{-7}$

Table S3. Binding energy for the obtained ten best poses of docking simulation.

POSES	ΔE(kcal/mol)
**1**	**-6.1**
2	-6.0
3	-5.8
4	-5.7
5	-5.7
6	-5.6
7	-5.6
8	-5.6
9	-5.5

a) H_4Iso

POSES	ΔE(kcal/mol)
**1**	**-6.1**
2	-6.0
3	-5.8
4	-5.7
5	-5.7
6	-5.6
7	-5.6
8	-5.5
9	-5.4

b) H_3Iso^-

Table S4. Tables of 10 structures obtained by RMSD-based clustering of MD simulations. Mpro : H_4Iso(top) and Mpro H_3Iso^- (down). From first column: cluster name, number of frames included in the cluster, population respect to total processed frames in percentage, average distance between frames inside the cluster expressed in Å, the centroid frame and the standard deviation related to AvgCDist.

a) H_4Iso

Cluster	Frames	Frac	AvgDist	Stdev	Centroid	AvgCDist
0	5149	52%	1,373	0,182	2614	1,697
1	2077	21%	1,243	0,212	7692	1,945
2	1193	12%	1,38	0,271	9787	1,854
3	472	5%	1,365	0,233	1136	1,833
4	357	4%	1,262	0,22	477	1,896
5	323	3%	1,297	0,25	271	1,952
6	149	2%	1,048	0,13	5841	1,885
7	140	1%	1,189	0,286	5629	1,953
8	89	1%	1,328	0	1366	1,901
9	51	1%	1,426	0	5233	1,869

b) H_3Iso^-

Cluster	Frames	Frac	AvgDist	Stdev	Centroid	AvgCDist
0	7524	75%	1,371	0,231	5444	2,374
1	852	9%	1,29	0,247	552	1,872
2	534	5%	1,191	0,175	2187	2,258
3	404	4%	1,245	0,243	1302	2,198
4	198	2%	1,212	0,198	1541	2,244
5	168	2%	1,249	0,231	1743	1,941
6	152	2%	1,343	0,254	128	2,047
7	80	1%	1,153	0,235	52	2,122
8	68	1%	1,087	0,134	1898	2,124
9	20	0%	0	0	772	2,062

Table S5 Results of MMPBSA calculations. Solvation free energies are calculated using the Poisson–Boltzmann (PB) implicit solvent method with a nonpolar solvation term, based on the solvent accessible surface area (SASA) present in AMBER16. The vibrational frequencies of normal modes are calculated at various local minima of the potential energy surface.[1]

a) H_4Iso

Energy Component	Average	Std. Dev.	Std. Err. Of Mean
VDWAALS	-27.87	3.18	0.32
EEL	-21.45	4.84	0.48
EPB	36.52	5.18	0.51
ENPOLAR	-2.82	0.14	0.01
EDISPER	0	0	0
DELTA G gas	-49.33	5.1	0,51
DELTA G solv	33.70	5.1	0.51
**DELTA TOTAL**	**-15.62**	3.35	0.33

b) H_3Iso^-

Energy Component	Average	Std. Dev.	Std. Err. Of Mean
VDWAALS	-20.86	3.88	0.38
EEL	87.05	16.73	1.66
EPB	-73.06	15.70	1.56
ENPOLAR	-2.29	0.29	0.03
EDISPER	0	0	0
DELTA G gas	66.19	14.66	1.46
DELTA G solv	-75.34	15.77	1.57
**DELTA TOTAL**	**-9.15**	3.01	0.29

[1] Case, D. A.; Ben-Shalom, I. Y.; Brozell, S. R.; Cerutti, D. S.; Cheatham, T. E., III; Cruzeiro, V. W. D.; Darden, T. A.; Duke, R. E.; Ghoreishi, D.; Gilson, M. K.; Gohlke,H.; Goetz, A. W.; Greene, D.; Harris, R.; Homeyer, N.; Izadi, S.; Kovalenko, A.; Kurtzman, T.; Lee, T. S.; LeGrand, S.; Li, P.; Lin, C.; Liu, J.; Luchko, T.; Luo, R.; Mermelstein, D. J.; Merz, K. M.; Miao, Y.; Monard, G.; Nguyen, C.; Nguyen, H.; Omelyan, I.; Onufriev, A.; Pan, F.; Qi, R.; Roe, D. R.; Roitberg, A.; Sagui, C.; Schott-Verdugo, S.; Shen, J.; Simmerling, C. L.; Smith, J.; Salomon-Ferrer,

R.; Swails, J.; Walker, R. C.; Wang, J.; Wei, H.; Wolf, R. M.; Wu, X.; Xiao, L.; York, D. M.; Kollman, P. A.AMBER 2017; University of California, San Francisco, 2017.
